# Low-Profile Dual-Polarized Double-Layer Microstrip Antenna for 5G and 5G Wi-Fi

**DOI:** 10.3390/mi14050942

**Published:** 2023-04-26

**Authors:** Wenxing An, Xiaoqing Tian, Jian Wang, Shuangshuang Wang

**Affiliations:** 1 Tianjin Key Laboratory of Imaging and Sensing Microelectronic Technology, School of Microelectronics, Tianjin University, Tianjin 300072, China; anwenxing@126.com (W.A.); 2020232173@tju.edu.cn (X.T.); 2 Qingdao Key Laboratory of Marine Information Perception and Transmission, Qingdao Institute of Ocean Engineering, Tianjin University, Qingdao 266200, China; 3 Tianjin Engineering Center of Integrated Circuit and Computing Systems, Tianjin 300072, China; 18526459322@163.com; 4 Tianjin International Joint Research Center of Internet of Things, Tianjin 300072, China

**Keywords:** microstrip antenna, double-layer, low-profile, wideband, periodical structure, dual-polarized

## Abstract

A dual-polarized double-layer microstrip antenna with a metasurface structure is proposed for 5G and 5G Wi-Fi. A total of 4 modified patches are used for the middle layer structure, and 24 square patches are used for the top layer structure. The double-layer design has achieved −10 dB bandwidths of 64.1% (3.13 GHz~6.08 GHz) and 61.1% (3.18 GHz~5.98 GHz). The dual aperture coupling method is adopted, and the measured port isolation is more than 31 dB. A low profile of 0.096λ_0_ is obtained (λ_0_ is the wavelength of 4.58 GHz in the air) for a compact design. Broadside radiation patterns have been realized, and the measured peak gains are 11.1 dBi and 11.3 dBi for two polarizations. The antenna structure and E-field distributions are discussed to clarify its working principle. This dual-polarized double-layer antenna can accommodate 5G and 5G Wi-Fi simultaneously, which can be a competitive candidate for 5G communication systems.

## 1. Introduction

The popularity of intelligent wireless terminals, autopilot, Virtual Reality (VR), and the Internet of Things (IoT) has put forward high requirements on wireless communication networks in terms of large system capacity, high transmission speed, and low network delay. Compared with 4th Generation (4G) wireless communication technology, 5th Generation (5G) technology can achieve a high-speed data rate with reduced network delay. It can carry out stable transmission in different scenarios. Recently, 5G wireless systems have been deployed widely. In addition, 5G WiFi has been developed for wireless environments inside buildings to improve indoor wireless network coverage. It has strong anti-interference ability, broad bandwidth, high throughput, and scalability. Most 5G systems operate between 3.3 GHz and 5 GHz, and the 5G WiFi band is from 5.15 GHz to 5.85 GHz.

Antennas for 5G and 5G Wireless-Fidelity (Wi-Fi) can be applied in the fields of mobile communication for high-speed data transmission and low-latency communication services, vehicle networking to achieve safer and more intelligent transportation systems, industrial automation for large-scale IoT device connections and data transmission, and medical and health for real-time interaction between medical devices and cloud data.

Single-polarized antennas have been studied extensively [1,2,3,4,5,6,7,8]. However, polarization diversity antennas have several advantages over traditional single-polarized antennas. Firstly, they can provide better signal quality and reliability by reducing signal fading and interference caused by polarization mismatch, which is particularly important in urban or crowded environments with multiple signals. Secondly, polarization diversity antennas can improve the range of wireless communication systems by reducing the effects of multipath propagation. Multipath propagation occurs when signals are reflected off surfaces and arrive at the receiving antenna at different times and phases, causing distortion and interference. Using two antennas with different polarization orientations, the receiver can combine the signals from both antennas and effectively cancel out the interference caused by multipath propagation. Finally, polarization diversity antennas can increase the capacity of wireless communication systems by allowing multiple users to transmit and receive data simultaneously on the same frequency band without interfering with each other. Overall, polarization diversity antennas offer significant improvements in signal quality, reliability, range, and capacity compared to traditional single-polarization antennas, making them a popular choice for a wide range of wireless communication applications.

Many antennas with dual-polarization performance have been investigated widely, for example, dipole [9,10,11,12,13,14], patch [15,16,17,18,19,20], and slot designs [21,22,23]. Many dual-polarized antennas working at the 5G frequency band have been designed. For dipole designs, a pair of printed bent dipoles was introduced with a balun and parallel transmission line for wideband performance [9]. A bandwidth of 10.5% was obtained with a port isolation of 52 dB and cross-polarization of 40 dB. A dual-polarized fan-shaped dipole antenna was designed with a frequency range from 3.3 GHz to 4.2 GHz [10]. A dual-polarized dipole antenna was exploited in [11]; the metasurface structure was placed between the dipole and the ground for a low profile. A bandwidth greater than 29.5% was achieved with an isolation of 34 dB and a height of 0.096λ_0_. The bandwidth was extended by a metal plating antenna with a 3D printing process [12]. It consisted of two orthogonal polarized dipoles. The tested bandwidth was from 3.5 GHz to 5.1 GHz with the voltage standing wave ratios less than 1.5. The antenna height was 0.24λ_0_ and the isolation was better than 18 dB. A novel dual-polarized magneto-electric dipole was reported in [13] with dielectric substrate loading. The antenna structure included vertical short-circuit patches and horizontal planar dipoles. Based on this design, the antenna had an impedance matching bandwidth of 24.9% and a height of 0.15λ_0_. The bandwidth of the dual-polarized magneto-electric dipole antenna was further improved to 65.9% [14]. It had stable radiation patterns with a port isolation greater than 36 dB. However, the antenna had a relatively larger height of 0.24λ_0_.

For the patch designs, a single-ended, dual-polarized patch antenna was proposed in [15]. Although an extremely low profile of 0.06λ_0_ was realized, it had a limited bandwidth of 5.7% with a port isolation of 25.4 dB. A patch antenna was discussed in [16] with a hybrid feed structure. The −10 dB bandwidth was 14% and the isolation level between two ports was less than 40 dB. To expand the bandwidth, a differentially driven dual-polarized patch antenna was investigated [17]. A wide impedance matching frequency band of 17.2% was obtained from 3.17 GHz to 3.77 GHz with a low profile of 0.067λ_0_ (λ_0_ is the free-space wavelength at 3.5 GHz). It had a high isolation greater than 38.5 dB and a low cross-polarization level less than −33 dB. A dual-polarized patch antenna was exploited with an etched bowtie slot [18]. A 10 dB reflection coefficient bandwidth of 18.8% was realized for two polarizations with a port isolation less than 28.5 dB and a low profile of 0.08λ_0_. To further improve the working frequency band, microstrip antenna designs using stacked patches have emerged [19,20]. A two-layer stacked patch was utilized to optimize the bandwidth [19]. A fractional impedance bandwidth of 19% was realized with a port isolation of 35 dB. In [20], a differentially fed stacked patch antenna was proposed for base station application. The driven patch was excited by tuned slots, and the top parasitic patch was employed to enhance the impedance bandwidth. A broadband performance of 49.4% was achieved with a high isolation greater than 37 dB.

For the slot designs, a differentially fed dual-polarized slot antenna was proposed for base station application [21]. Two H-shaped slots were etched on an octagon patch. It had a bandwidth of 19.3% with a VSWR (voltage standing wave ratio) less than 1.5 and a port isolation larger than 43 dB. The frequency band was improved by a microstrip-fed stepped-impedance slot antenna [22]. The slot was excited by a stepped feeding strip. The tested relative bandwidth was 38.7% from 1.69 GHz to 2.5 GHz with an isolation greater than 35 dB. The frequency band was further widened by a dual-polarized cross-shaped slot antenna, which was excited by U-shaped microstrip lines [23]. A broad bandwidth of up to 68% was achieved with an |*S*_11_| less than −10 dB from 1.3 GHz to 2.65 GHz. However, the port isolation was only 20 dB, and the profile was 0.27λ_0_.

There are also some other dual-polarized designs. A folded bowtie antenna was discussed in [24] operating between 3.1 and 5 GHz with a relatively large profile. The filtering antenna had a wide working band of 25.6% and a low cross-polarization ratio of 22 dB [25]. A differentially fed filtering antenna was reported for the 5G frequency band. It had dual operation bands from 3.28 GHz to 3.71 GHz and from 4.8 GHz to 5.18 GHz [26]. A wideband differentially fed laminated resonator antenna was reported in [27] with a wideband of 29% and a port isolation better than 35 dB.

In 2014, a broadband low-profile metasurface antenna was presented based on a periodical mushroom structure [28]. The proposed antenna was formed using 4 × 4 mushroom cells and a ground plane. The operating principle of the metasurface antenna was investigated, and the field distributions of the resonance modes were examined. Then, the metasurface antennas were studied extensively due to their desired features such as low-profile structure, stable radiation performance, high efficiency, and good cross-polarizations. Recently, metasurface antennas have been designed to accommodate different communication systems including 5G and 5G WiFi [29,30,31,32]. The bandwidth was extended to 28% in [29]. A dual-polarized metasurface antenna was reported in [30]. It had a low profile of 0.058λ_0_ with a −10 dB bandwidth of 25%, and the port isolation was greater than 34 dB. To improve the frequency band, a dual-polarized grid-slotted microstrip antenna was designed using a Y-shaped feeding strip [31]. The tested port isolation was better than 14.5 dB with a wideband performance of 43%. However, only part of the 5G band was covered. Then, a dual-polarized antenna with a low profile was designed for the 5G frequency band [32]. Its working band was from 3.2 GHz to 5.1 GHz with a bandwidth of 53.4%. However, only part of the 5G and 5G Wi-Fi bands were covered. So, some novel designs are expected to accommodate the 5G sub-6 GHz (3.3 to 5 GHz) and 5G Wi-Fi (5.15 GHz to 5.85 GHz) bands with a relative bandwidth of at least 55%. Furthermore, a low-profile design is more appreciated as it can save installation space effectively.

A comparison is conducted in Table 1 including different kinds of dual-polarized works. References [9,10,12,20,24] have relatively large profiles. References [13,21,22,25,26,27] have relatively narrow working frequency bands. Although references [11,15,16,17,18,19,29,30,31,32] have low-profile designs, their working frequency bands are relatively narrow. References [14,23] have broadband performances. However, the reported antenna heights were higher than 0.23λ_0_.

To achieve the required bandwidth, low profile, high isolation, and higher gain performance, we designed a dual-polarized low-profile broadband antenna to accommodate 5G and 5G Wi-Fi bands. The paper is organized as follows. The background of the dual-polarized antenna is discussed in Section 1. The detailed double-layer structure and materials of the proposed antenna elements are described in Section 2. To validate the dual-polarized design, a prototype with the metasurface was fabricated and tested in Section 3. The working mechanism, the influence of the double-layer structure, and the influence of the feeding strip on the antenna performance are investigated in Section 4. Some concluding remarks are given in Section 5.

## 2. Materials and Methods

**Figure 1:**
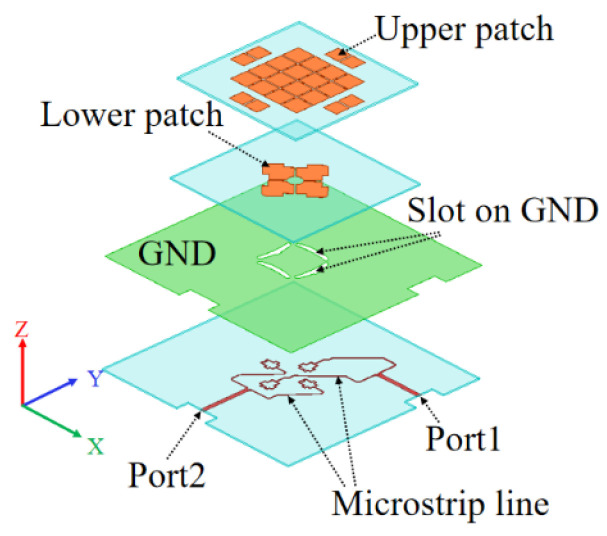
The proposed wideband dual-polarized double-layer antenna.

The proposed dual-polarized antenna is shown in Figure 1. The dual-polarized microstrip antennas consist of a double-layer metallic structure, a ground plane, and feeding strips printed on dielectric substrates. The double-layer patches are made of copper, and the substrates are typically made of insulating material. The ground plane serves as a reflector.

Relong and Epoxy glass fiber (FR4) laminates are employed for the top and middle layer substrates. The double-layer structure of the microstrip antennas has several advantages. It provides improved bandwidth and radiation patterns as the two layers can be designed to have different dielectric constants and thicknesses to achieve desired characteristics. The Relong and FR4 laminates have relative dielectric constants of 2.2 and 4.4, respectively. Their loss tangents are 0.0009 and 0.02. An air gap is used to separate the Relong and FR4 substrates. The use of low dielectric constant materials in microstrip antennas offers several advantages. Firstly, it can decrease the Q factor and improve the radiation efficiency of the antenna by reducing losses due to dielectric absorption. Secondly, it can increase the bandwidth of the antenna by reducing the effect of surface waves. So, low dielectric constant materials in microstrip antennas can improve the antenna’s performance. Detailed antenna structural parameters are in Table 2.

**Figure 2:**
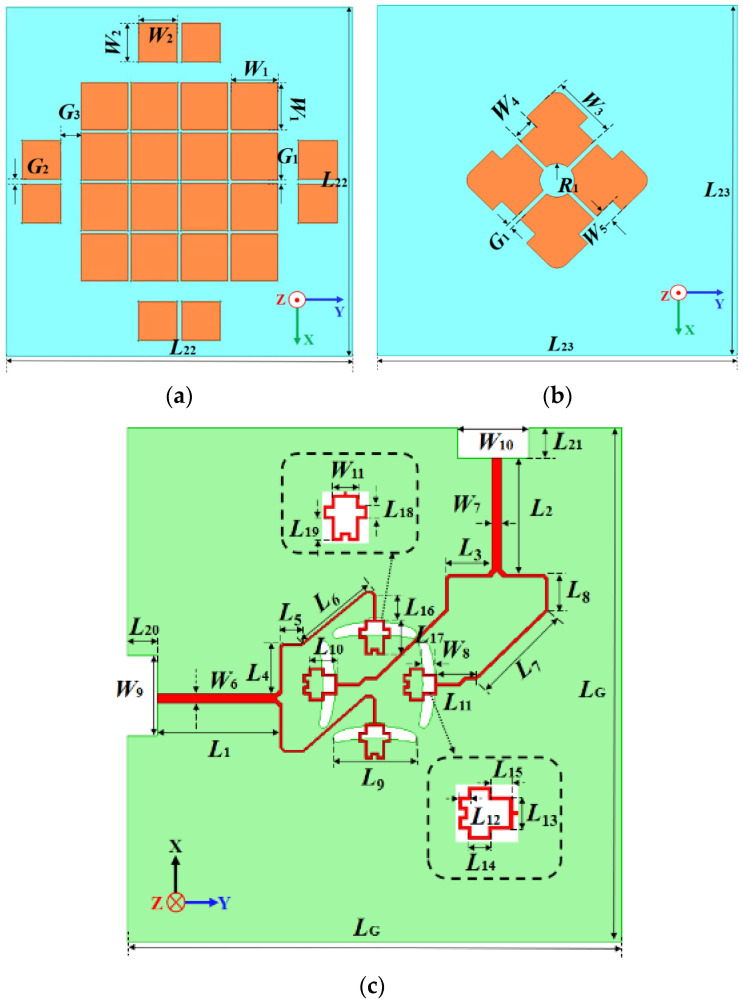
The dual-polarized antenna structure: (**a**) top structure; (**b**) middle layer; (**c**) ground plane.

The double-layer structure is at the center of the ground with a size of 125 × 125 mm^2^. The top layer has a height of 5.3 mm, and the adopted Relong substrate has a size of 80 × 80 mm^2^ with a thickness of 1 mm. In Figure 2a, 16 square patches are printed at the center while 8 square patches are at the four edges for better impedance matching, especially at the higher band. The central patches have a separation of *G*_1_ with a length of *W*_1_. The edge patch has a length of *W*_2_ with a gap of *G*_2_. The middle layer is placed above the ground with a height of 2 mm. In Figure 2b, there are four metallic patches at the center of the 0.8 mm thick FR4 substrate with a size of 83 × 83 mm^2^. The patch shape is optimized with a circular slot at the center.

In Figure 2c, the feed networks are placed at the bottom of the 31 mil thick Relong substrate. An aperture-coupled feeding method is adopted for the antenna excitation, which can decrease the current discontinuity on the patches. An aperture-coupled feeding technique has several advantages over other feeding methods. Firstly, aperture-coupled feeding provides a simple and compact structure for easy integration with other circuits and components. The feeding structure consists of a microstrip line with a slot coupled to the radiating element. The microstrip line can connect other circuits or components, such as filters or amplifiers, which makes it a versatile and flexible feeding method for various applications. Secondly, the coupling between the microstrip line and the radiating element can be adjusted by varying the gap size, providing more flexibility for a wider operating bandwidth compared to other feeding methods, such as coaxial or waveguide feeding. Overall, aperture-coupled feeding can offer a simple, compact, and high-performance feeding method for antenna design, making it a popular choice in many applications.

For each polarization in this design, two arc-shaped slots are carved on the ground, stimulated by the feedline on the bottom of this substrate. The feeding strip intersection would result in low port isolation [31]. A power divider is introduced into the feed structure, and the microstrip feedlines for two polarization directions are properly designed to avoid the intersection [30,32]. Good port isolation is achieved across the passband. The terminals of the feedline structure are modified for better impedance matching, which will be discussed later.

## 3. Results

The double-layer antenna prototype was fabricated and assembled to verify the proposed antenna design. HFSS (High-Frequency Structure Simulator) software was adopted for antenna simulation and optimization. HFSS is a powerful 3D electromagnetic simulation software widely used in the design and analysis of high-frequency electronic components and systems. It uses the finite element method (FEM) to analyze the electromagnetic characteristics of three-dimensional objects. An air box was used to surround the antenna model. By setting radiation boundary conditions on the surface of an air box, an infinite space can be simulated. HFSS software uses adaptive mesh generation technology to automatically generate accurate and effective meshes to complete the discretization of analysis objects. Usually, the grid size is less than one-tenth of the wavelength corresponding to the solution frequency.

**Figure 3:**
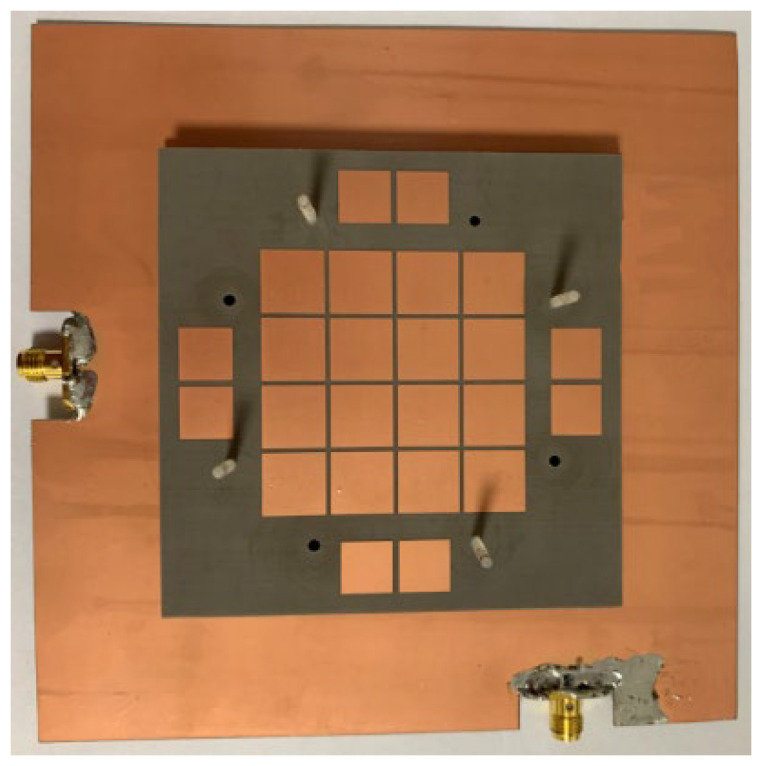
Antenna prototype.

The antenna prototype is in Figure 3. The fabrication procedure was as follows: Firstly, the multilayer antenna structure was fabricated using printed circuit board technology. Then, the SMA connector was welded to the feed port on the antenna ground. Finally, the multilayer antenna structures were assembled, and the middle and upper structures were fixed on the ground at specific heights using plastic screws.

**Figure 4:**
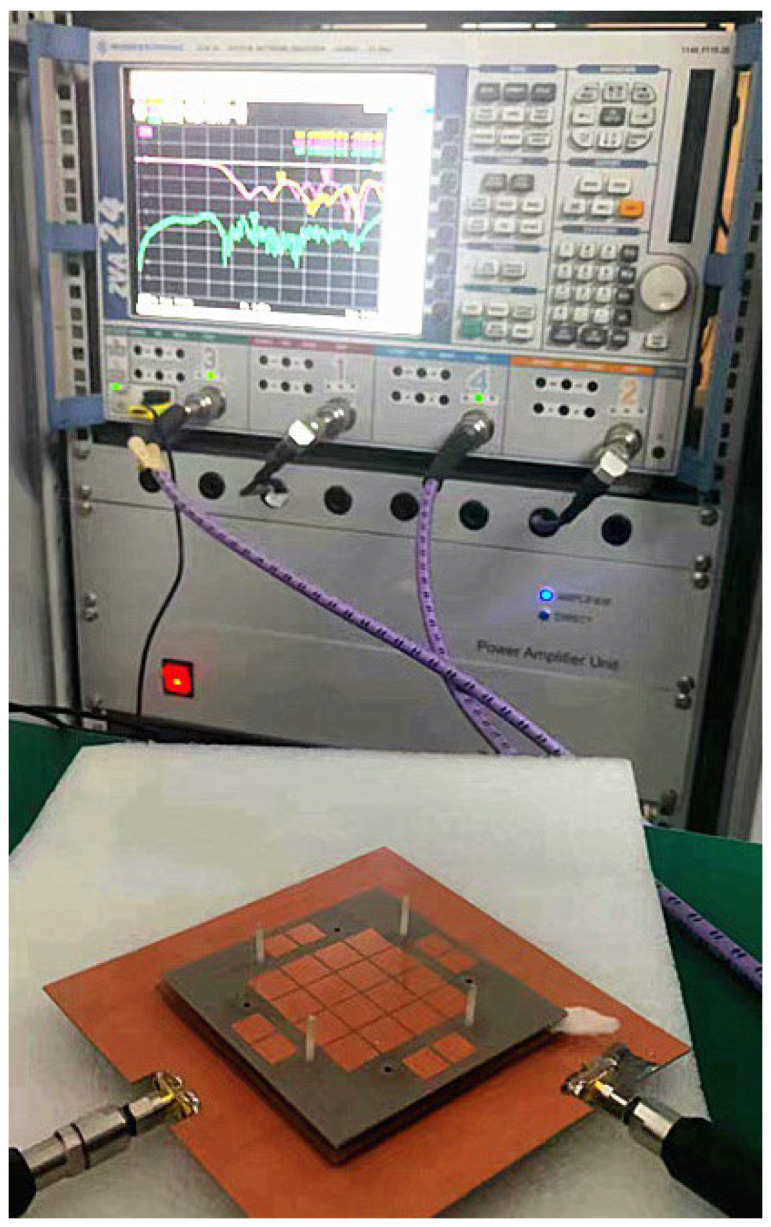
Antenna prototype testing scenario with Rohde & Schwarz ZVA24 network analyzer.

**Figure 5:**
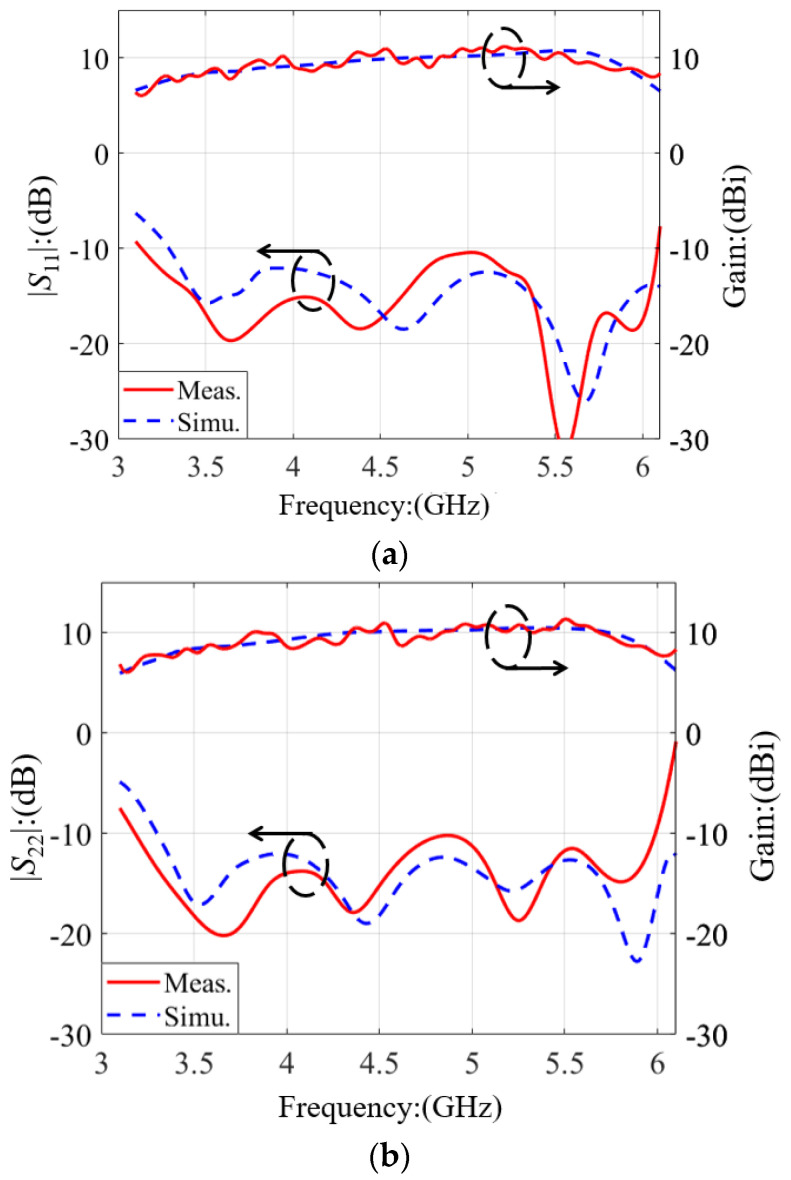
Measured and simulated *S*-parameters and gains: (**a**) Port 1; (**b**) Port 2.

A Rohde & Schwarz ZVA24 network analyzer was used for the measurement of the S-parameter, as shown in Figure 4. The parameters of the Rohde & Schwarz ZVA24 network analyzer are in Table 3. Two ports of the dual-polarized antenna were connected to the network analyzer, and the tested S-parameter results were measured and exported. Figure 5 depicts the calculated and tested results of the |*S*_11_|, |*S*_22_|, |*S*_12_|, and gain. Although there were some assembly errors, the test and simulation results are consistent. For Port 1, the calculated frequency band less than −10 dB started from 3.29 GHz, and the tested band was from 3.13 GHz to 6.08 GHz with a relative bandwidth of 64.1%. For Port 2, the calculated band started from 3.3 GHz, and the measured band was from 3.18 GHz to 5.98 GHz with a relative bandwidth of 61.1%. Based on the tested results, the frequency band between 3.3 GHz and 5.875 GHz was completely covered.

**Figure 6:**
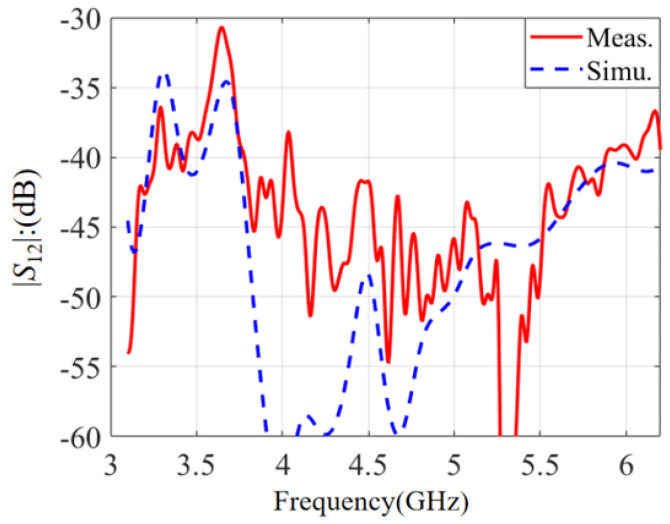
Calculated and tested port isolation.

The calculated and tested port isolations are plotted in Figure 6. Due to the assembly errors, there are certain deviations between the simulated and tested results. Though the tested |*S*_12_| is slightly different from the simulation result, the tested |*S*_12_| can be maintained below −31 dB using the optimized feeding network. So, satisfactory port isolation has been accomplished for the dual-polarized antenna.

**Figure 7:**
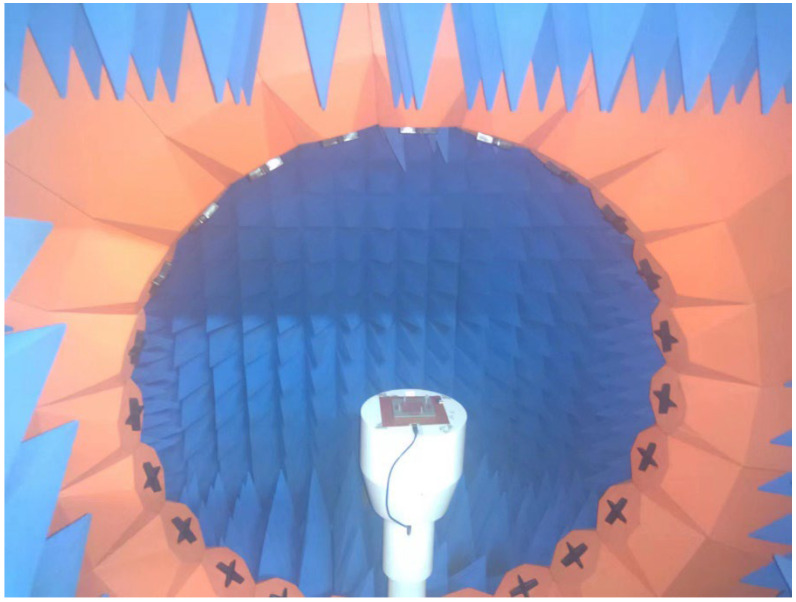
Antenna prototype testing scenario in the chamber.

The radiation performance was obtained in an anechoic chamber. An ATS200 multi-probe test system with a spherical near-field (SNF) testing method was used to measure the radiation properties of the antenna [33]. The near-field testing environment had 23 near-field probes in Figure 7. Its performance parameters are in Table 4. The antenna was placed at the center of the spherical measurement range, and the near-fields were tested at a series of points on a sphere enclosing the antenna. The SNF method is used to measure the far-field radiation properties of an antenna, which cannot be directly measured in a laboratory environment. The technique involves measuring the electromagnetic field on a spherical surface surrounding the antenna; then, an algorithm is used to convert the measurements into a far-field radiation pattern. The advantages of the SNF method include its ability to measure the far-field radiation properties of an antenna in a laboratory environment and its accuracy in measuring complex radiation patterns. The method is widely used in the design and testing of antennas for various applications.

The tested gain performances are also plotted in Figure 5. For the 5G and 5G Wi-Fi bands between 3.3 GHz and 5.875 GHz, the measured gain of Port 1 varied between 7.9 dBi and 11.1 dBi. The average value was 9.62 dBi with a tested peak value of 11.3 dB at 5.2 GHz. The measured gain of Port 2 fluctuated between 7.7 dBi and 11.3 dBi. The average value was 9.64 dBi with a measured peak value of 11.3 dB at 5.5 GHz.

**Figure 8:**
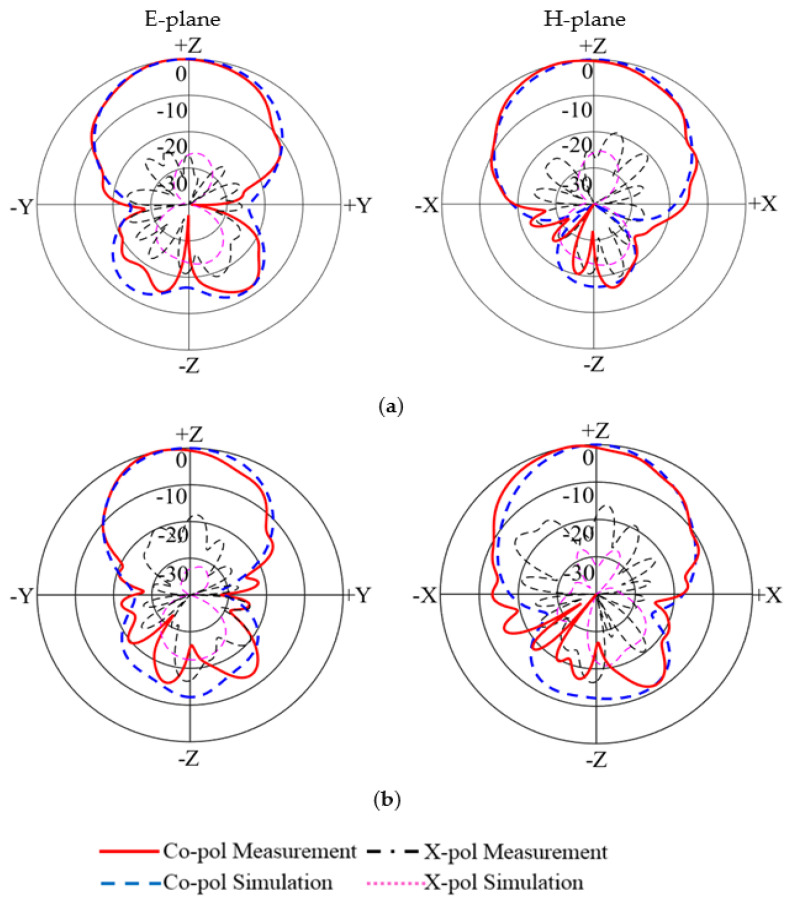
Calculated and tested radiation patterns of Port 1: (**a**) 4 GHz; (**b**) 5 GHz.

**Figure 9:**
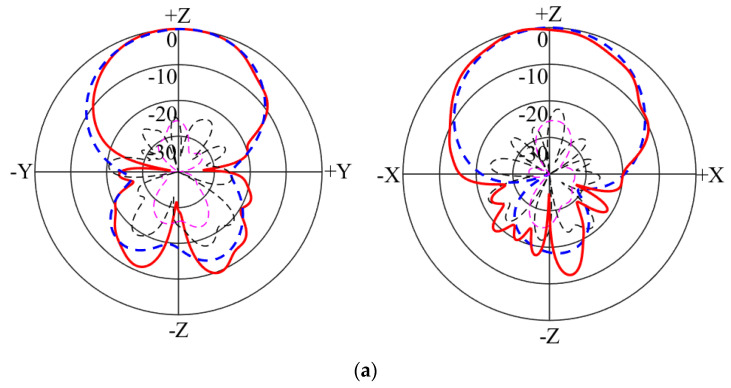

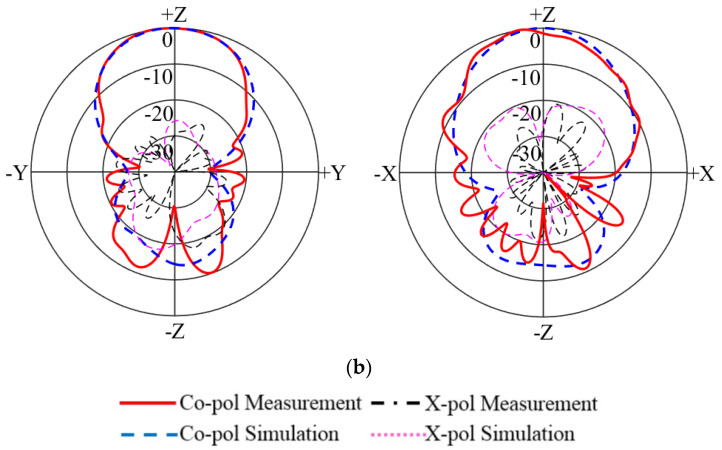
Calculated and tested radiation patterns of Port 2: (**a**) 4 GHz; (**b**) 5 GHz.

The calculated and tested radiation patterns of the two polarizations are plotted in Figure 8 and Figure 9 at 4 GHz and 5 GHz. It is noticed that broadside radiation patterns have been achieved across the passband. The calculated and tested co-polarization curves almost overlap with each other. The tested cross-polarizations at 4 GHz and 5 GHz were less than −14.8 dB for Port 1 and −18 dB for Port 2. The measured back radiation levels at 4 GHz and 5 GHz were less than −11 dB for Port 1 and −10 dB for Port 2. So, low cross-polarization and back-lobe levels have been obtained.

## 4. Discussions

The antenna’s structures were discussed to clarify their influence on the antenna’s performance, including the top layer and feedline. Many theories have been developed for antenna mode analysis; for example, the eigenmode [30] and characteristic mode [34]. In this design, the electric field distributions were analyzed to investigate the working mechanism. Port 2 was selected for the following discussion.

**Figure 10:**
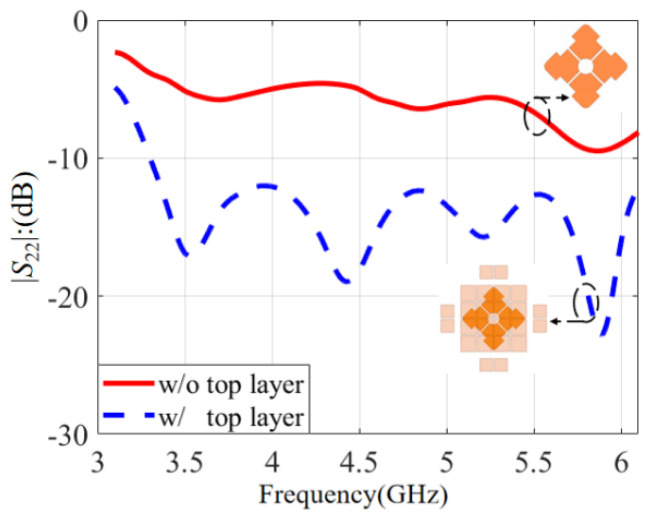
The influence of the top layer on the *S*-parameter.

Firstly, the influence of the top layer’s structure on the antenna performance will be discussed. The comparison of the *S*-parameters with and without the top layer is conducted in Figure 10. It is clear that the top layer had a significant effect on impedance matching. The curve of |*S*_22_| without the top layer was above −10 dB. So, the antenna with only the middle layer had poor impedance matching. After loading the top layer structure, the |*S*_22_| was improved effectively, and the curve was below −10 dB. So, a top layer structure can improve impedance matching effectively. It was concluded that the middle layer and the ground formed the basic microstrip structure and the top layer was loaded to augment the antenna’s performance.

**Figure 11:**
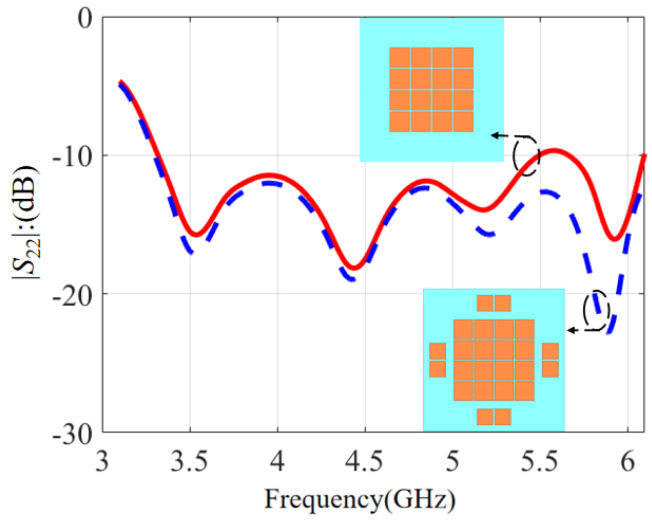
The influence of edge patches on the *S*-parameter.

The *S*-parameters with and without edge patches are compared in Figure 11. The red solid line is based on 16 patches while the blue dashed line is based on the proposed design with 8 edge patches. It is noticed that the impedance matching with 16 patches deteriorated slightly near 5.5 GHz and the |*S*_22_| was barely maintained at −10 dB. After loading the eight edge patches, the impedance at the higher frequency became better and the |*S*_22_| was less than −12 dB across the passband. So, edge patches can improve impedance matching at a higher band effectively, and a wider bandwidth can be accomplished.

**Figure 12:**
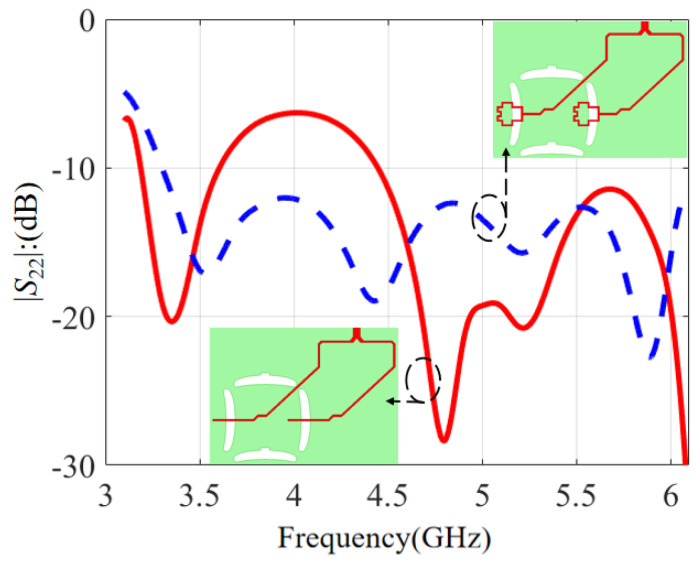
The influence of different feeding strips on the *S*-parameter.

The *S*-parameters with different feeding strips are compared in Figure 12. The red solid line is with a common straight feeding strip, while the blue dashed line is based on the feeding strip with a modified terminal structure. It is observed that the |*S*_22_| with an ordinary straight feeding strip was only −7 dB near 4 GHz. To optimize the working frequency band, the feeding strip structure was modified with an optimized terminal structure. Then, the impedance matching near 4 GHz was improved effectively and the |*S*_22_| became better than −12 dB across the passband. So, a modified feeding strip can improve the S-parameter at 4 GHz.

**Figure 13:**
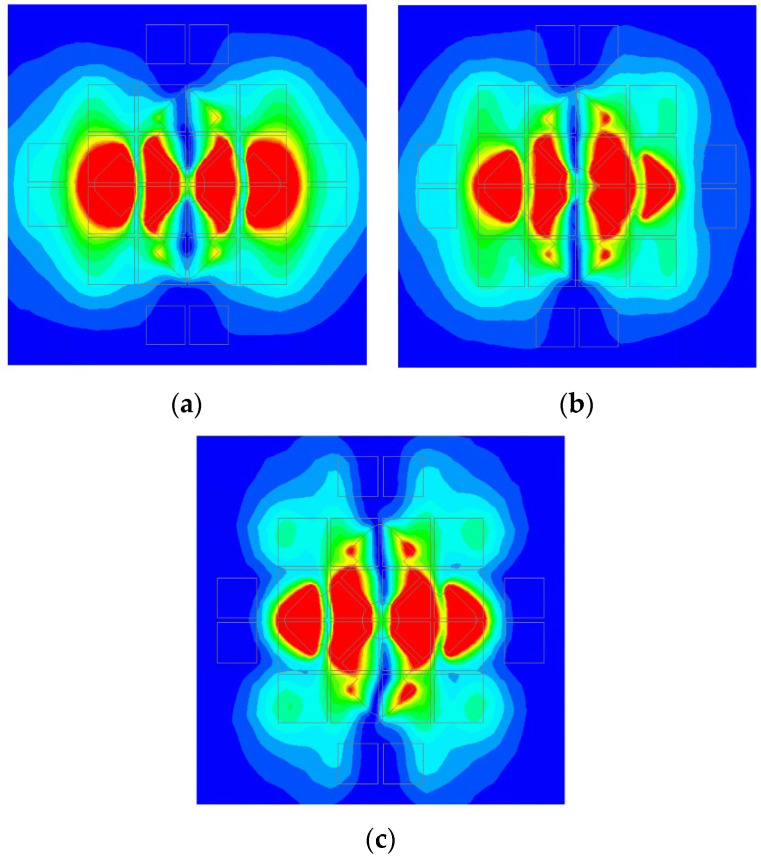
Effective electric field distributions of the microstrip antenna: (**a**) 3.5 GHz; (**b**) 4.5 GHz; (**c**) 5.5 GHz.

**Figure 14:**
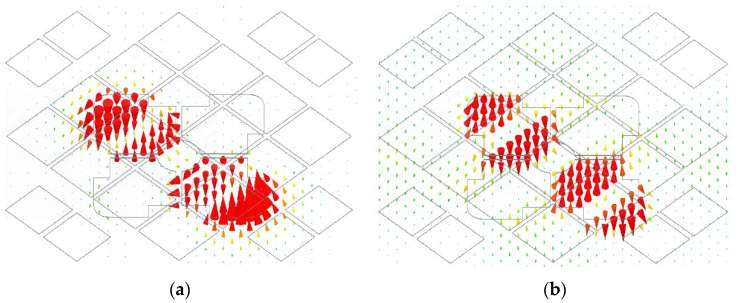

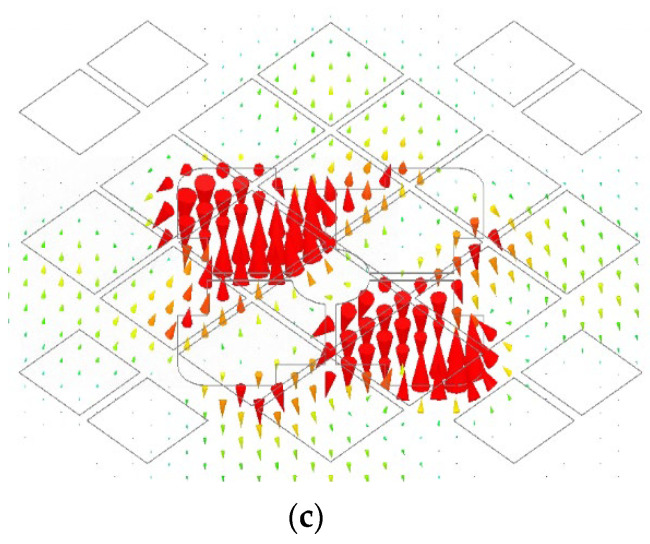
Instantaneous electric field distributions of the proposed antenna: (**a**) 3.5 GHz; (**b**) 4.5 GHz; (**c**) 5.5 GHz.

To analyze the working mechanism of this dual-polarized antenna, effective and instantaneous electric field distributions between the radiating patches and the ground are plotted in Figure 13 and Figure 14 with the electric field in the horizontal direction. Three frequency points were selected at 3.5 GHz, 4.5 GHz, and 5.5 GHz. The eigenmode method was adopted to discuss its working principle. It is observed from Figure 13 that the effective electric field distributions were symmetrical with four maximum points along the *Y*-axis. As the antenna had a large resonant structure at a low frequency, the effective electric field area was also relatively large in the polarization direction. When the frequency increased, the effective electric field area was reduced in the polarization direction.

Corresponding to the effective electric field distribution, the instantaneous electric field is plotted in Figure 14. It is noticed that the electric field direction changed three times in the polarization direction at three frequencies. Because of these separated patch structures, the electric field phase changed by 180 degrees at the center in the polarization direction. Based on the electric field distribution, the proposed double-layer microstrip antenna was mainly in anti-phase TM_20_ mode at low, middle, and high frequencies. So, wideband performance was obtained based on the proposed structure.

## 5. Conclusions

A dual-polarized double-layer antenna was investigated for 5G and 5G Wi-Fi. The middle layer and the ground constructed the basic antenna structure, and a metasurface structure was applied for the top layer to improve impedance matching. Broadband performances of 64.1% (3.13 GHz~6.08 GHz) and 61.1% (3.18 GHz~5.98 GHz) were achieved with anti-phase TM_20_ modes across the passband. A feeding strip with a modified terminal structure can improve the impedance matching of the middle band, and parasitic edge patches can optimize the impedance matching of the higher band effectively. A high port isolation of 31 dB was achieved. Furthermore, the antenna had a low profile of 0.096λ_0_ for space saving and easy installation. The antenna element had realized high peak gains of 11.1 dB and 11.3 dB with broadside radiation patterns and low cross-polarizations. With these favorable characteristics, this broadband dual-polarized antenna should find widespread applications for 5G and 5G Wi-Fi communications.

## Figures and Tables

**Table 1:** Comparison of this work with other dual-polarized designs.

Ref.	Height (λ_0_ @ Center Freq.)	Relative Bandwidth (%)	Port Isolation (dB)	Element Peak Gain (dBi)	Ant. Type
[9]	0.25λ_0_	10.5	52	9.9	dipole
[10]	0.26λ_0_	24	25	7	dipole
[11]	0.096λ_0_	29.5	20	9	dipole
[12]	0.24λ_0_	37.2	18	9	dipole
[13]	0.15λ_0_	24.9	29	8.2	dipole
[14]	0.23λ_0_	65.9	36	9.5	dipole
[15]	0.06λ_0_	5.71%	24	10.9	patch
[16]	0.096λ_0_	14	40	7.4	patch
[17]	0.067λ_0_	17.2	38.5	8.2	patch
[18]	0.08λ_0_	18.8	28.5	8	patch
[19]	0.08λ_0_	19	35	11	patch
[20]	0.27λ_0_	49.4	37	8.7	patch
[21]	0.13λ_0_	19.3	43	8.1	slot
[22]	0.16λ_0_	38.7	35	N.A.	slot
[23]	0.27λ_0_	68	20	9	slot
[24]	0.45λ_0_	46.9	28	7.5	folded bowtie
[25]	0.14λ_0_	25	20	N.A.	filtering ant.
[26]	0.13λ_0_	12.3 and 7.6	37	8.34	filtering ant.
[27]	0.115λ_0_	29.2	35	7.58	laminated ant.
[30]	0.058λ_0_	25	34	10	metasurface
[31]	0.06λ_0_	43	14.5	10.1	metasurface
[32]	0.047λ_0_	53.4	30	9.9	metasurface

**Table 2:** Antenna parameters (mm).

Para.	Value	Para.	Value	Para.	Value	Para.	Value	Para.	Value	Para.	Value	Para.	Value
*L_G_*	125.0	*L*_6_	22.0	*L*_12_	2.0	*L*_18_	2.5	*W*_1_	10.5	*W*_7_	2.4	*G*_1_	1.1
*L*_1_	31.0	*L*_7_	24.2	*L*_13_	5.0	*L*_19_	4.0	*W*_2_	8.7	*W*_8_	3.0	*G*_3_	4.6
*L*_2_	28.2	*L*_8_	9.0	*L*_14_	3.8	*L*_20_	7.5	*W*_3_	15.0	*W*_9_	19.5	*R*_1_	4.0
*L*_3_	11.8	*L*_9_	20.1	*L*_15_	3.4	*L*_21_	7.5	*W*_4_	5.0	*W*_10_	18.0		
*L*_4_	12.2	*L*_10_	7.1	*L*_16_	6.0	*L*_22_	80.0	*W*_5_	3.0	*W*_11_	5.0		
*L*_5_	4.8	*L*_11_	10.0	*L*_17_	8.5	*L*_23_	83.0	*W*_6_	2.3	*G*_1_	0.8		

**Table 3:** The performance parameters of the Rohde & Schwarz ZVA24 network analyzer.

Parameter	Performance
Frequency range	67 GHz
Port	4
Dynamic range	>150 dB
Output power	>18 dBm
Measurement speed	<2 μs
Sample time	430 ns
IF bandwidth	15 MHz

**Table 4:** The performance parameters of the multi-probe test system.

Parameter	Performance
Frequency range	800 MHz~6 GHz
Dynamic range	75 dB
Gain stability	0.3 dB
TRP stability	0.5 dB
TIS stability	0.7 dB
Polarization	Circular, linear, elliptical

## Data Availability

Not applicable.
